# Supplementary material for: Direct observation of chaperone-modulated talin mechanics with single-molecule resolution
Source: Commun Biol. 2022 Apr 4;5:307. doi: 10.1038/s42003-022-03258-3 (PMC8979947; doi:10.1038/s42003-022-03258-3)
Supplement: Supplementary file 2 — Supplementary Information [file 42003_2022_3258_MOESM2_ESM.pdf]

Supplementary Information

Direct observation of chaperone-modulated talin mechanics with single molecule resolution

Soham Chakraborty[1†], Deep Chaudhuri[1†], Souradeep Banerjee[1†], Madhu Bhatt[1], Shubhasis Haldar[1]*

[1]Department of Biological Sciences, Ashoka University, Sonepat, Haryana, India

[†]These authors contributed equally

*to whom correspondence may be addressed. Email- shubhasis.haldar@ashoka.edu.in

Supplementary Figure 1: Hysteresis percentages in force-ramp experiments with different chaperones.

Supplementary Figure 2: Rupture/unfolding forces of talin at varying loading rates.

Supplementary Figure 3: One-way ANOVA analysis of talin unfolding force with different chaperones.

Supplementary Figure 4: One-way ANOVA analysis of talin refolding force with different chaperones.

Supplementary Figure 5: Representative trace of talin R3-IVVI in the presence of 1 µM DnaJ.

Supplementary Figure 6: Representative trace of talin in presence of 3 µM apo-DnaK.

Supplementary Figure 7: Representative trace of talin in presence of DnaKJ-ATP complex.

Supplementary Figure 8: Representative trace of talin domain in the presence of DnaKJE-ATP complex.

Supplementary Figure 9: Representative trace of talin R3-IVVI in presence of 60 µM DsbA.

Supplementary Figure 10: Fraction folded of talin with different chaperones at varying forces.

Supplementary Figure 11: Chevron plots with the different nucleotide states of DnaK chaperone.

Supplementary Figure 12: Unfolding and refolding rates with different chaperones.

Supplementary Figure 13: Unfolding and refolding rates with DnaK+GrpE at different nucleotide states.

Supplementary Figure 14: Intersection force of talin WT-R3.

Supplementary Figure 15: Talin folding dynamics in presence of Hsp70 and Hsp40

Supplementary Figure 16: SDS-PAGE of protein samples.

Supplementary Figure 17: Magnet law.

Supplementary Methods: Force calibration method

Testing the accuracy of force calibration

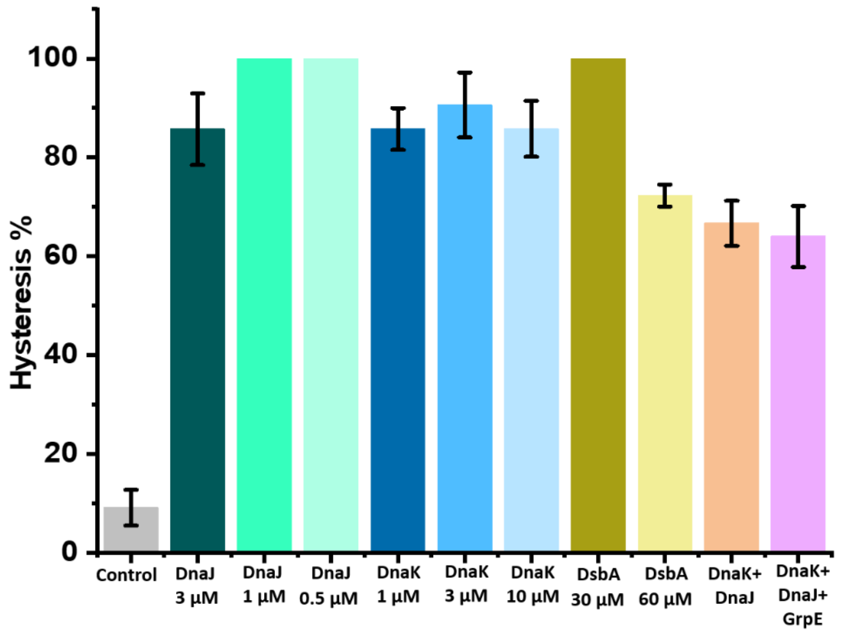

Supplementary Figure 1: Hysteresis percentages in force-ramp experiments with different chaperones. *We observed that hysteresis is substantially increased in the presence of chaperones. In the absence of chaperones or in control, the hysteresis is observed in ~9% events, while it increases to ~86% and ~91% upon the addition of DnaJ and DnaK, respectively. This hysteresis could occur due to altered binding dynamics in molecular interaction where smaller hysteresis could denote rapid interaction kinetics and the larger hysteresis mean slow interaction kinetics. The mechanical unfolding of protein molecules during a force-ramp experiment is performed under non-equilibrium conditions where the complex tertiary contacts of the protein break and requires a slower time-scale to equilibrate. This time-scale for equilibration might become further slower during the chaperone interaction, possibly resulting in the more pronounced hysteric nature in the force-extension curve. Here hysteresis does not significantly affect the determination of unfolding and refolding steps as these steps are much clear and easy to detect. We studied more than 50 events in each case to check the hysteresis.*

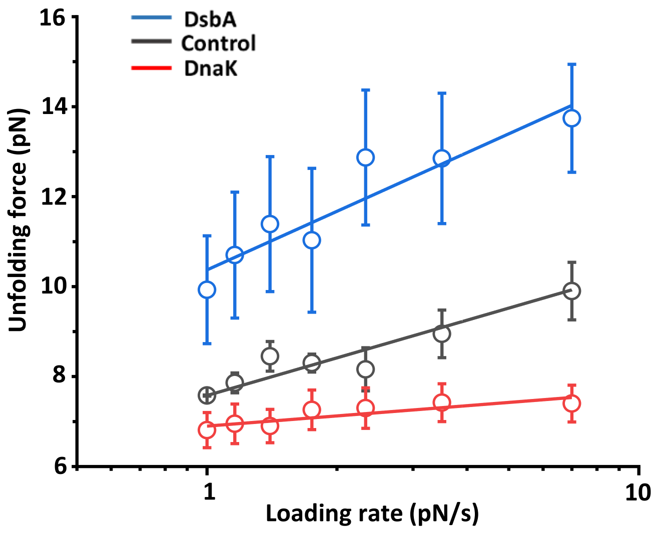

Supplementary Figure 2: Rupture/unfolding forces of talin at varying loading rates. *The unfolding forces are plotted as a function of varying loading rates, ranging from 1 to 7 pN/s. The unfolding forces at zero loading rate has been observed to change in the presence of different chaperones, signifying the change in mechanical stabilities of talin with different chaperones. For example, in the absence of any chaperones, the unfolding force is 7.6±0.01 pN, which has been observed to shift to 6.9±0.1 pN with DnaK and increases to 10.4±0.3 pN with DsbA as foldase. This signifies the increased mechanical stability of talin upon DsbA interaction and lower stability with DnaK unfoldase chaperone. Data points are measured using more than three individual molecules at each loading rate. Error bars are s.e.m.*

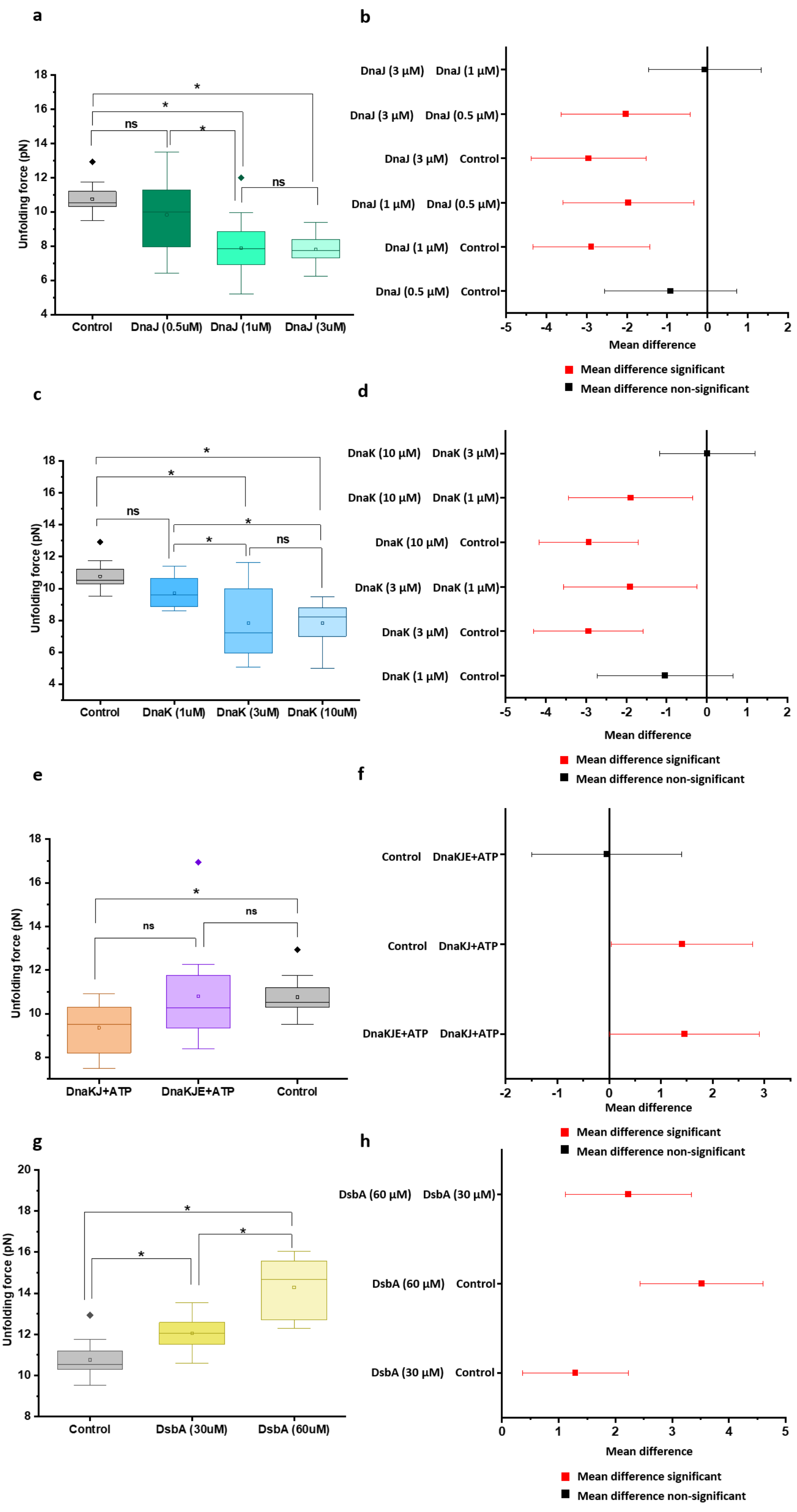
a
b
c
d
e
f
g
h
Unfolding force (pN)
Control
DnaJ (0.5uM)
DnaJ (1uM)
DnaJ (3uM)
ns
*
DnaJ (3 µM) DnaJ (1 µM)
DnaJ (3 µM) DnaJ (0.5 µM)
DnaJ (3 µM) Control
DnaJ (1 µM) DnaJ (0.5 µM)
DnaJ (1 µM) Control
DnaJ (0.5 µM) Control
Mean difference
Mean difference significant
Mean difference non-significant
DnaK (1uM)
DnaK (3uM)
DnaK (10uM)
DnaK (10 µM) DnaK (3 µM)
DnaK (10 µM) DnaK (1 µM)
DnaK (10 µM) Control
DnaK (3 µM) DnaK (1 µM)
DnaK (3 µM) Control
DnaK (1 µM) Control
DnaKJ+ATP
DnaKJE+ATP
Control DnaKJE+ATP
Control DnaKJ+ATP
DnaKJE+ATP DnaKJ+ATP
DsbA (30uM)
DsbA (60uM)
DsbA (60 µM) DsbA (30 µM)
DsbA (60 µM) Control
DsbA (30 µM) Control

Supplementary Figure 3: One-way ANOVA analysis of talin unfolding force with different chaperones. ***(a** and **b)** ANOVA analysis has been performed to check the statistical significance of unfolding force with different concentrations of DnaJ. We observed that the unfolding force becomes saturated at 1µM, and thus the mean force differences become non-significant (ns) between 1 and 3 µM concentrations at *p≤0.05 level (R sq. = 0.456). We further performed the Bonferroni post-hoc test to check the mean comparisons with different DnaJ concentrations.* ***(c** and **d)** Similarly, we performed the ANOVA analysis for different DnaK concentrations and found that the unfolding force becomes saturated at 3 µM and thus showed non-significance between 3 and 10 µM concentrations (R sq. = 0.454 at *p≤0.05).* ***(e** and **f)** Unfolding force with DnaKJ is statistically significant with both DnaKJE and control (R sq. = 0.184), which is also evident in post-hoc analysis.* ***(g** and **h)** The unfolding force in the absence of DsbA (control) is significantly different from 60 µM DsbA (R sq. = 0.670), which is shown in the Bonferroni test. For each chaperone data set, unfolding force are measured. For Control, n=15; DnaJ, n =10 (0.5 µM); n= 16 (1 µM), n= 17 (3 µM); DnaK, n =8 (1 µM); n= 17 (3 µM), n= 28 (10 µM); DnaKJ, n =15; DnaKJE, n=12; DsbA, n=13 (30 µM), n=8 (60 µM).*

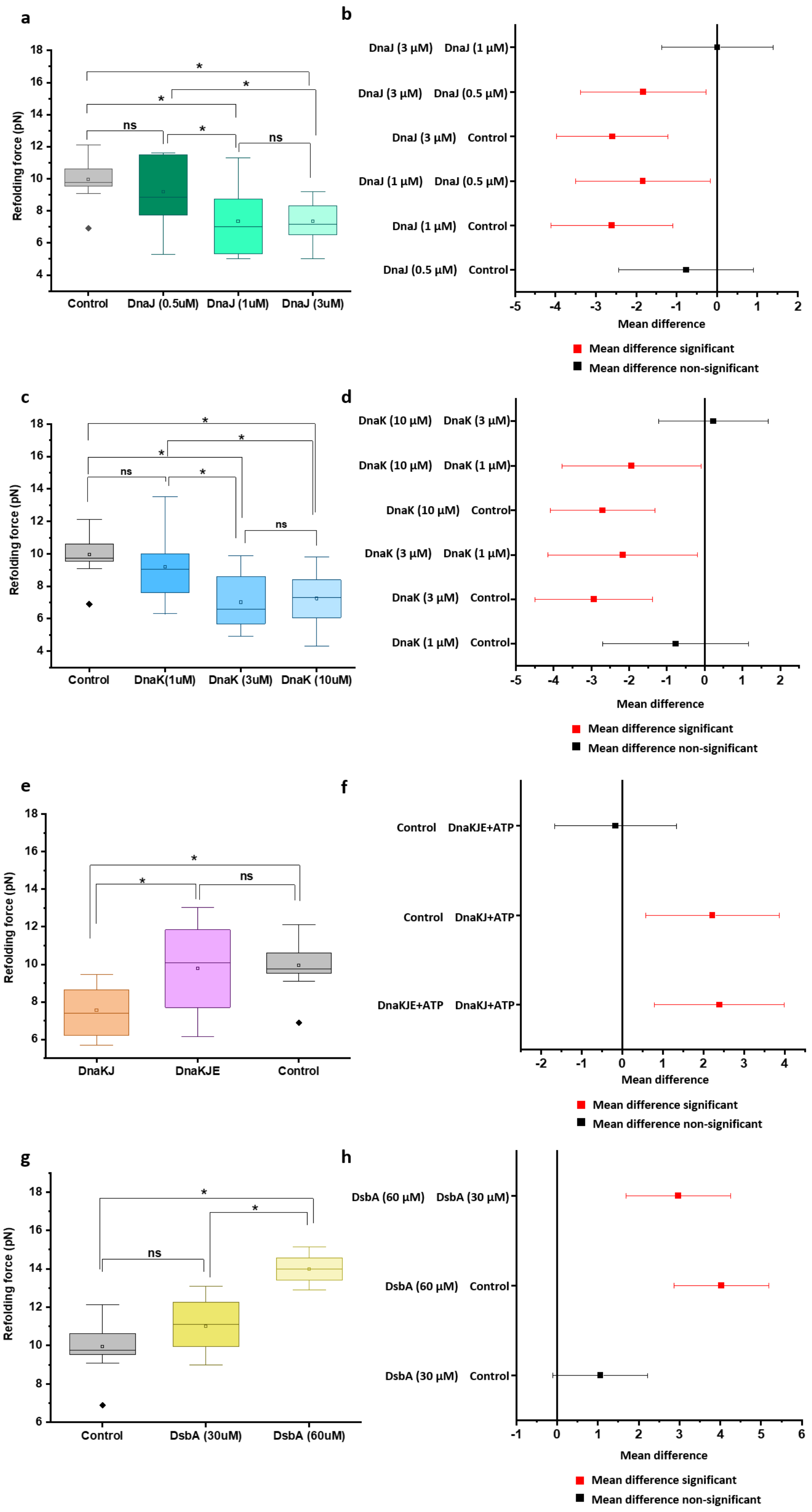
a
Refolding force (pN)
Control
DnaJ (0.5uM)
DnaJ (1uM)
DnaJ (3uM)
ns
*
b
DnaJ (3 µM) DnaJ (1 µM)
DnaJ (3 µM) DnaJ (0.5 µM)
DnaJ (3 µM) Control
DnaJ (1 µM) DnaJ (0.5 µM)
DnaJ (1 µM) Control
DnaJ (0.5 µM) Control
Mean difference
Mean difference significant
Mean difference non-significant
c
Refolding force (pN)
Control
DnaK(1uM)
DnaK (3uM)
DnaK (10uM)
d
DnaK (10 µM) DnaK (3 µM)
DnaK (10 µM) DnaK (1 µM)
DnaK (10 µM) Control
DnaK (3 µM) DnaK (1 µM)
DnaK (3 µM) Control
DnaK (1 µM) Control
Mean difference
Mean difference significant
Mean difference non-significant
e
Refolding force (pN)
DnaKJ
DnaKJE
Control
f
Control DnaKJE+ATP
Control DnaKJ+ATP
DnaKJE+ATP DnaKJ+ATP
Mean difference
Mean difference significant
Mean difference non-significant
g
Refolding force (pN)
Control
DsbA (30uM)
DsbA (60uM)
h
DsbA (60 µM) DsbA (30 µM)
DsbA (60 µM) Control
DsbA (30 µM) Control
Mean difference
Mean difference significant
Mean difference non-significant

***Supplementary Figure 4: One-way ANOVA analysis of talin refolding force with different chaperones. (a** and **b)** ANOVA analysis has been performed to check the statistical significance of refolding force at different concentrations of DnaJ. We observed that the refolding force becomes saturated at 1-3 µM concentration, and thus the mean force differences become non-significant at *$p \leq 0.05$ level (R sq. = 0.363). We further performed the Bonferroni post-hoc test to check the mean comparison with different DnaJ concentrations. **(c** and **d)** Similarly, we performed the ANOVA analysis for different DnaK concentrations and found that the refolding force becomes saturated at 3µM and thus showed non-significance between 3 and 10 µM concentration (R sq. = 0.414 at *$p \leq 0.05$). **(e** and **f)** Refolding force with DnaKJ is statistically significant with both DnaKJ and control (R sq. = 0.297), which is also evident in post-hoc analysis. **(g** and **h)** The refolding force in the absence of DsbA (control) is significantly lower from 60 µM DsbA (R sq. = 0.703), which is shown in Bonferroni post-hoc test. For each chaperone data set, refolding force are measured and averaged. For Control, n=16; DnaJ, n =11 (0.5 µM); n= 16 (1 µM), n= 24 (3 µM); DnaK, n =7 (1 µM); n= 14 (3 µM), n= 23 (10 µM); DnaKJ, n =11; DnaKJE, n=14; DsbA, n=10 (30 µM), n=10 (60 µM).*

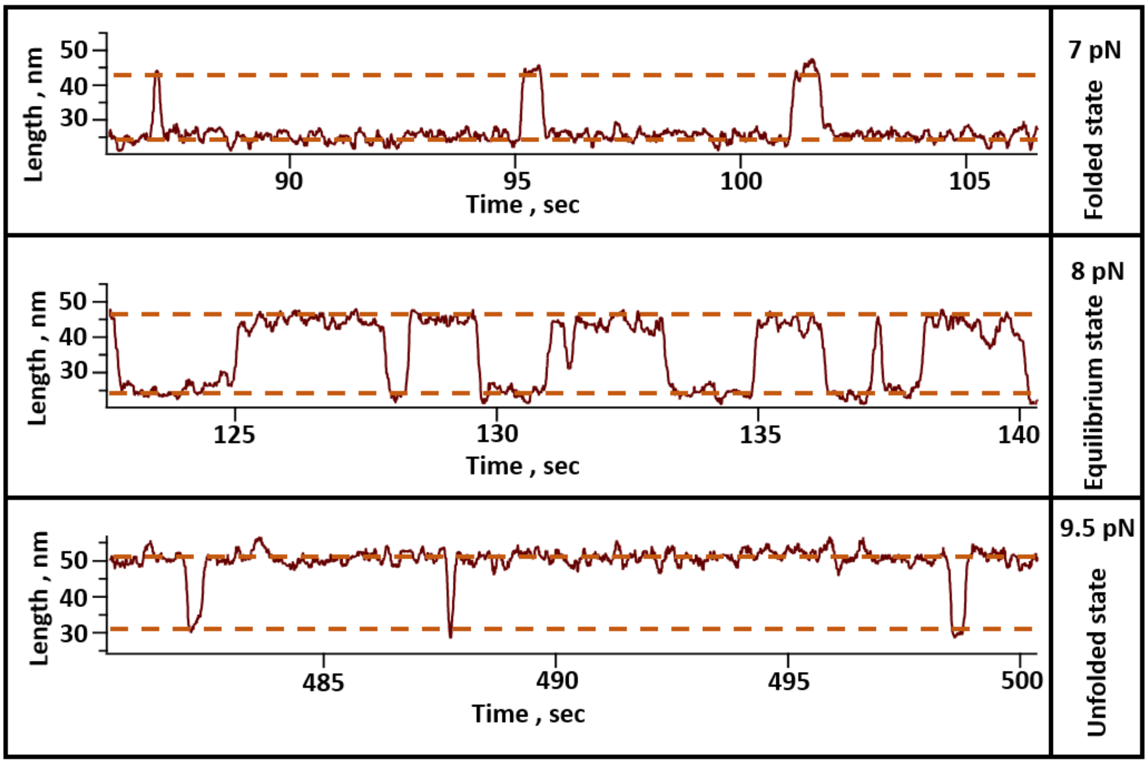

Supplementary Figure 5: Representative trace of talin R3-IVVI in the presence of 1 µM DnaJ. *In the presence of 1 µM DnaJ, the folding dynamics of talin domain shifts towards the lower force regime. At 7 pN, talin mostly stays in the folded state (top trace) and at 9.5 pN force, it mostly stays in the unfolded state. At 8 pN, the domain occupies both the folded and unfolded states.*

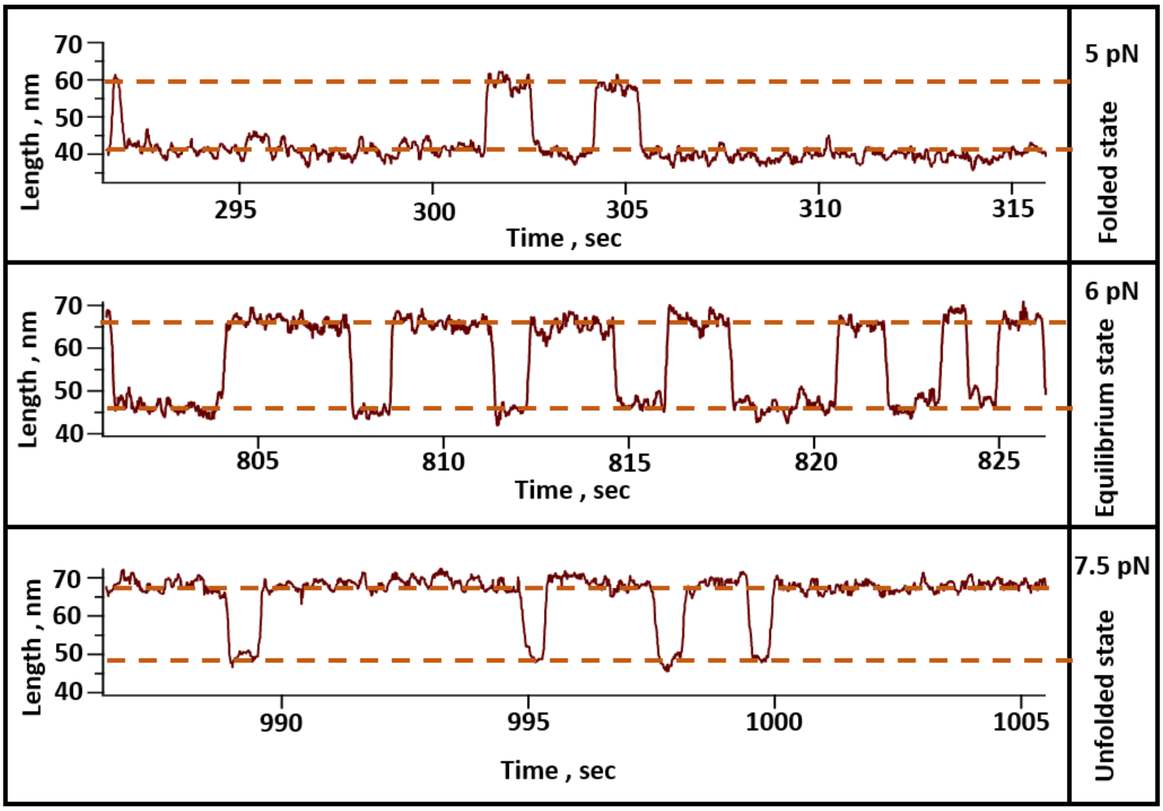

Supplementary Figure 6: Representative trace of talin in presence of 3 µM apo-DnaK. *In the presence of 3 µM DnaK without any nucleotide, the folding dynamics of talin shifts towards the lower force regime. The domain mostly stays in the folded state at 5 pN (top trace), whereas, at 7.5 pN it mostly populates in the unfolded state (bottom trace). At 6 pN force, both the folded and unfolded states are almost equally populated in the domain.*

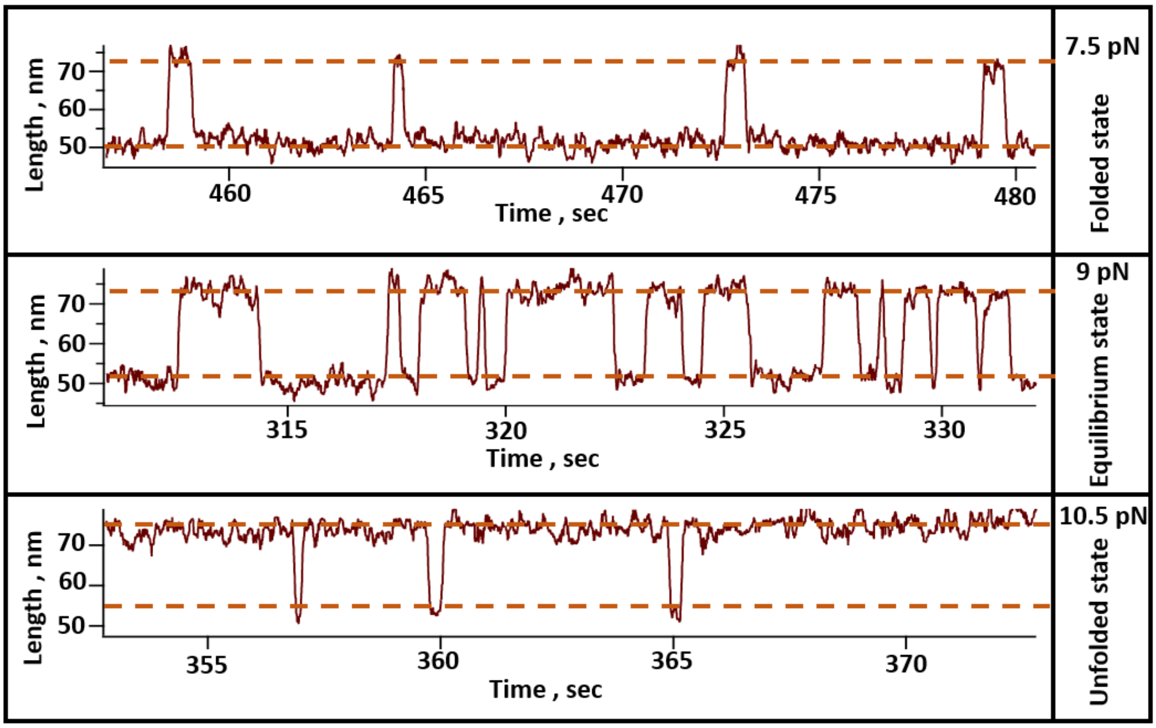

Supplementary Figure 7: Representative trace of talin in presence of DnaKJ-ATP complex. *We monitored the folding dynamics of talin R3-IVVI in the presence of 1 µM DnaJ, 3 µM DnaK and 10 mM ATP and 10 mM* $MgCl_2$*. The buffer is exchanged after every 30 minutes with fresh ATP to keep the sufficient supply of ATP. In this condition, talin mostly populates in the folded state at 7.5 pN (top trace) and mostly in the unfolded state at 10.5 pN (bottom trace). At 9 pN, both the folded and unfolded states are equally populated.*

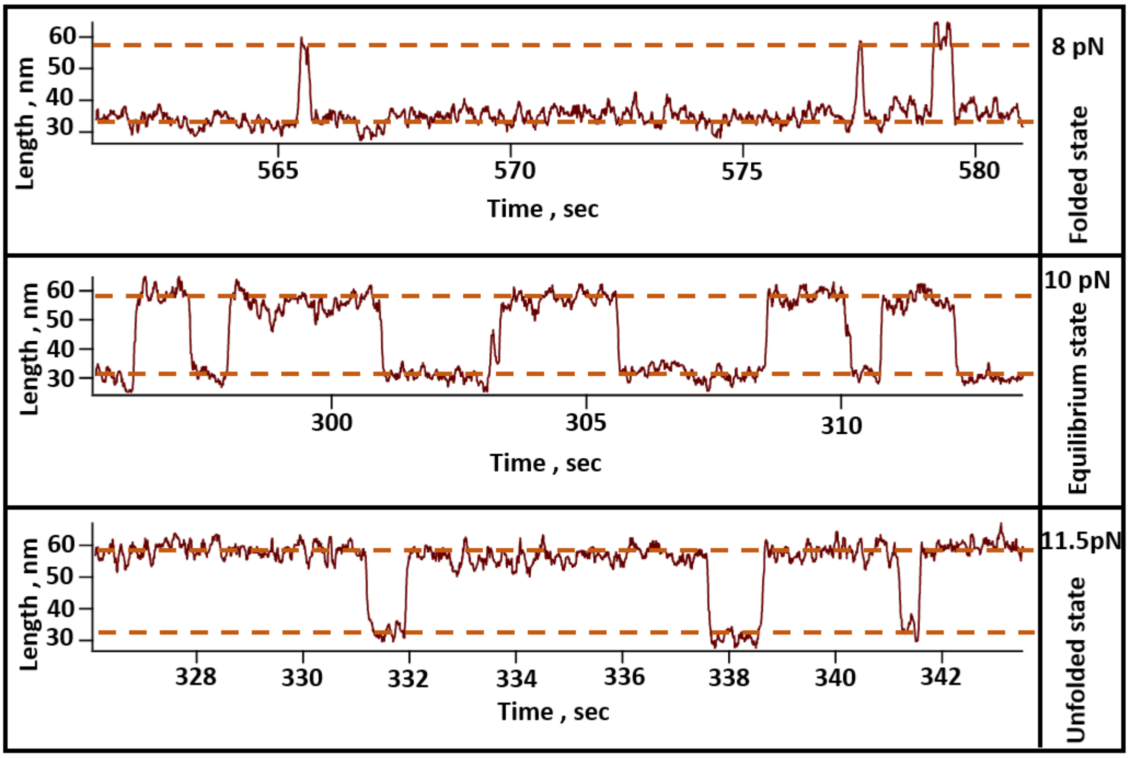

Supplementary Figure 8: Representative trace of talin domain in the presence of DnaKJE-ATP complex. *The dynamics of talin has been monitored in the presence of 1µM DnaJ, 3µM DnaK, 5 µM GrpE and 10 mM ATP and 10 mM* $MgCl_2$*.The buffer is exchanged after every 30 minutes with fresh ATP to maintain the sufficient supply of ATP. DnaKJE complex restores the ability of folding dynamics in R3-IVVI and this domain mostly stays in the folded state at 8 pN (top trace) and in unfolded at 11.5 pN (bottom trace).*

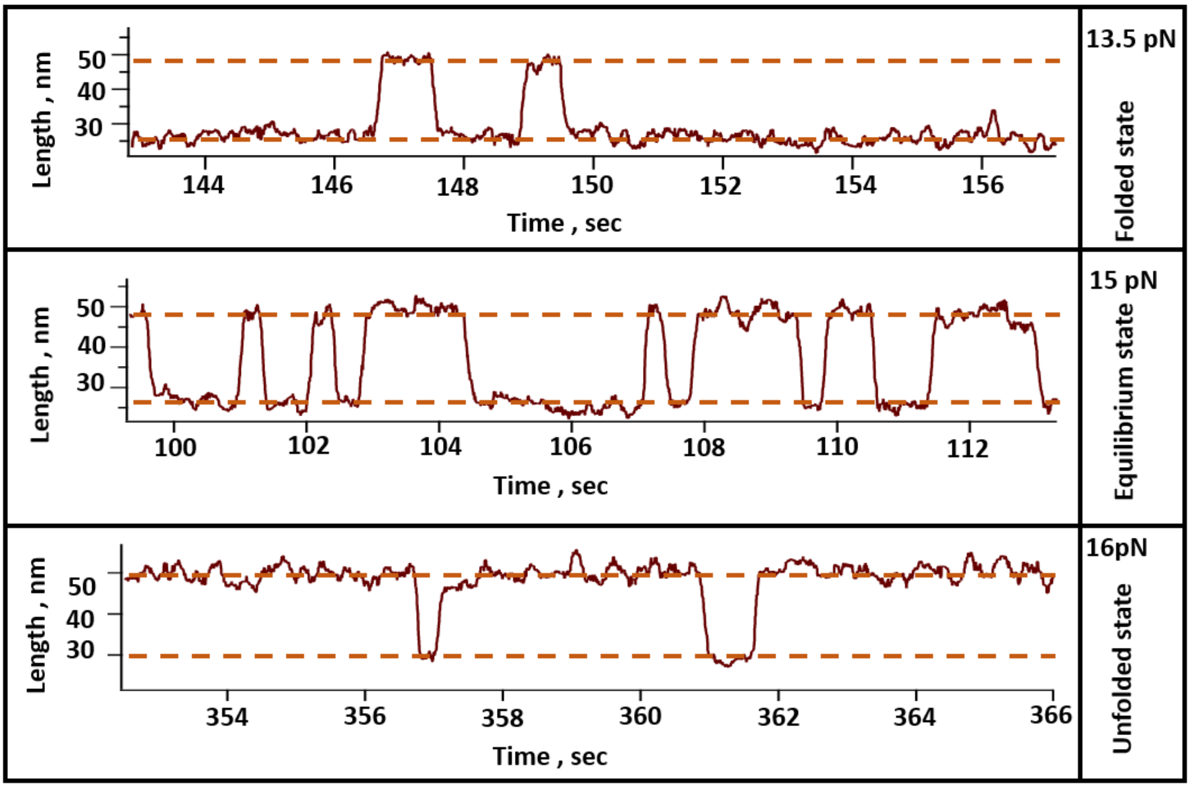

Supplementary Figure 9: Representative trace of talin R3-IVVI in presence of 60 µM DsbA. *In the presence of 60 µM DsbA, the folding dynamics of talin R3-IVVI domain shifts towards the higher force regime. The domain mostly stays in the folded state at 13.5 pN force (top trace), whereas at 16 pN force, it mostly populates in the unfolded state (bottom trace). At 15 pN force, both the folded and unfolded states are equally populated.*

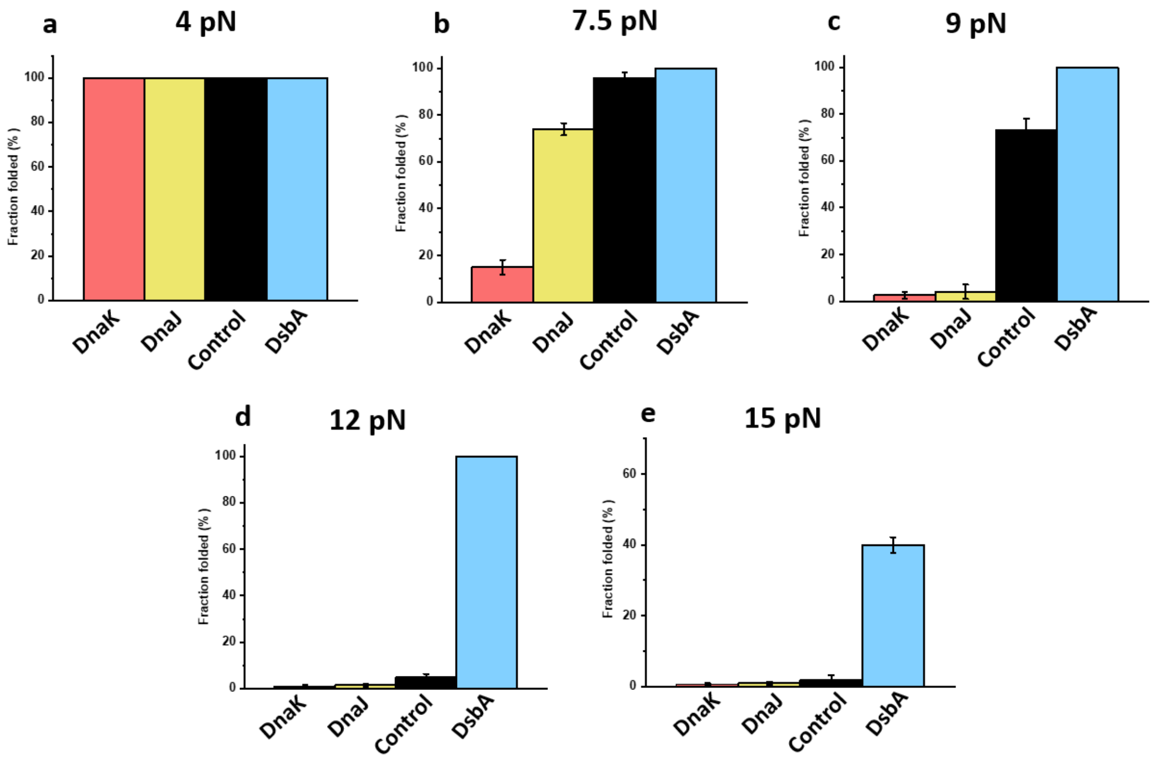

Supplementary Figure 10: Fraction folded of talin with different chaperones at varying forces. a. *At 4 pN force, the fraction force of talin remains 100%, irrespective to chaperone presence.* ***(b*** *and* ***c)*** *however, the chaperones effect become more prominent at intermediate forces of 7.5 and 9 pN, where the folded fraction decreases significantly with two unfoldases. For example, at 9 pN, the fraction folded with DnaK and DnaJ are 2.6±1.5 % and 4±3.1 %, respectively; while remains 100% with DsbA due to its foldase activity, however, in control (in the absence of any chaperones), the fraction percentage decreases to only 73±5.12.* ***(d)*** *Since DsbA acts as a strong foldase under force, it significantly increases the talin stability by shifting the half-point force, and thus,* ***(e)*** *the domain still could remain folded at 15 pN (40±2.22 %) in the presence of DsbA, while loses its ability either in control or with unfoldases.*

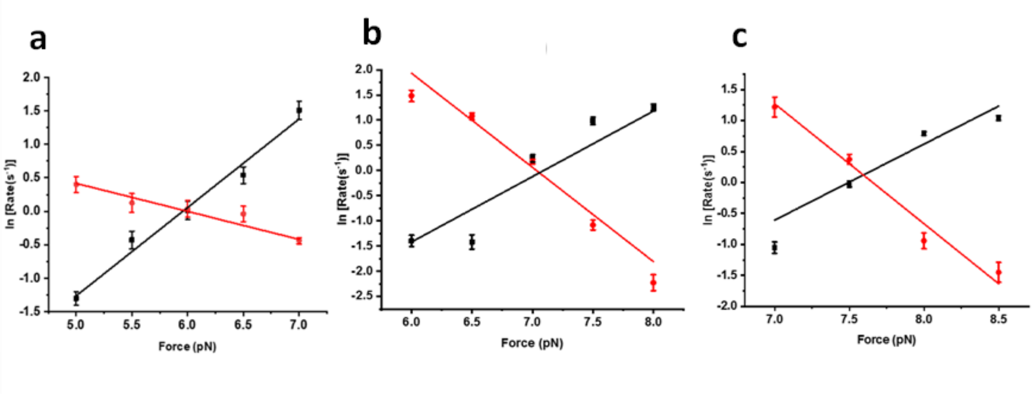

Supplementary Figure 11: Chevron plots with the different nucleotide states of DnaK chaperone. (a) Apo-DnaK: *In nucleotide-free state or in apo-state of DnaK, the intersection force of talin is 6 pN.* ***(b) DnaK-ADP:*** *with DnaK-ADP complex, the intersection force of talin is 7.1 pN.* ***(c) DnaK-ATP:*** *in DnaK-ATP state, the intersection force of talin is 7.6 pN. Data points are calculated by averaging minimum three molecules per force. The errors are relative error of log.*

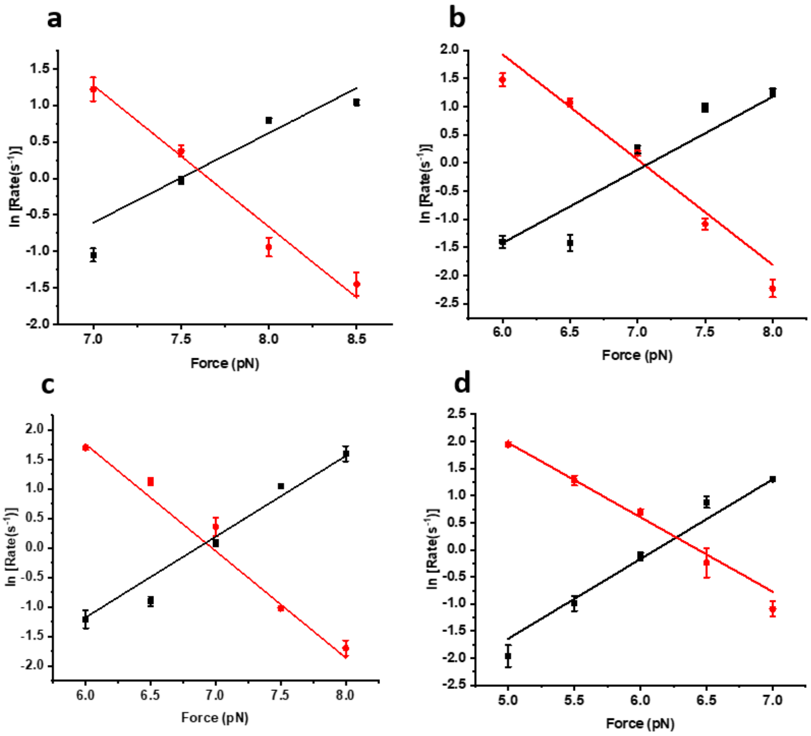

Supplementary Figure 12: Unfolding and refolding rates with different chaperones. *The unfolding and refolding kinetics are plotted as a function of force, in the presence different nucleotide states of DnaK-GrpE. Unfolding rate increases and refolding rate decreases with the force and the cross-point of these two rates is defined as intersection force.* ***(a) DnaK +ATP:*** *In the presence of DnaK+ATP, the intersection force is 7.6 pN. Error bars are relative error of log.* ***(b) DnaK +ADP:*** *With DnaK+ADP, the intersection force has been observed to decrease from 9.7 pN to 7.1 pN. Error bars are relative error of log.* ***(c) DnaKJ+ATP:*** *Similarly, with DnaKJ+ATP complex, the intersection force of talin is 6.9 pN. Error bars are relative error of log.* ***(d) DnaKJ+ADP:*** *The intersection force decreases further to 6.3 pN with*

DnaKJ+ADP complex. Data points are calculated using minimum three molecules per force. Error bars are relative error of log.

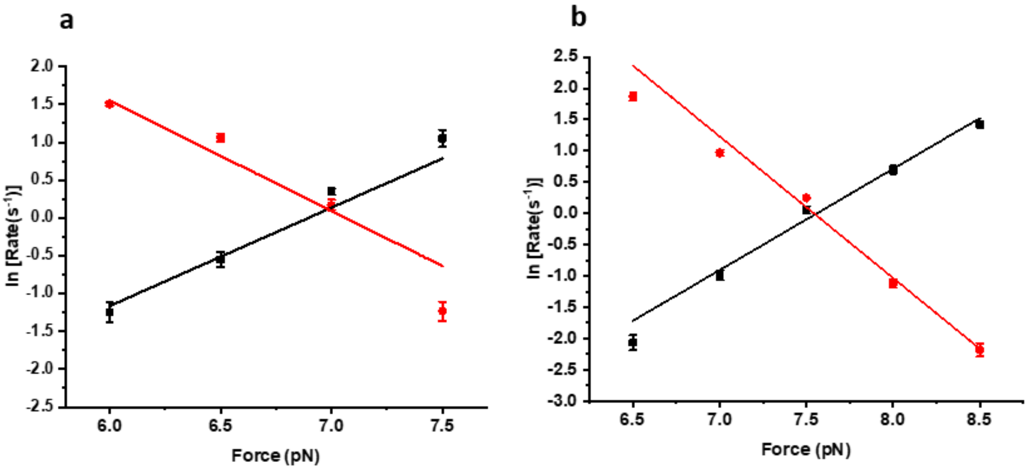

Supplementary Figure 13: Unfolding and refolding rates with DnaK+GrpE at different nucleotide states. (a) KE-ADP: *the intersection force is 6.9 pN.* ***(b) KE-ATP:*** *the intersection force is 7.5 pN. Data points are calculated using four individual molecules per force. Error bars are relative error of log.*

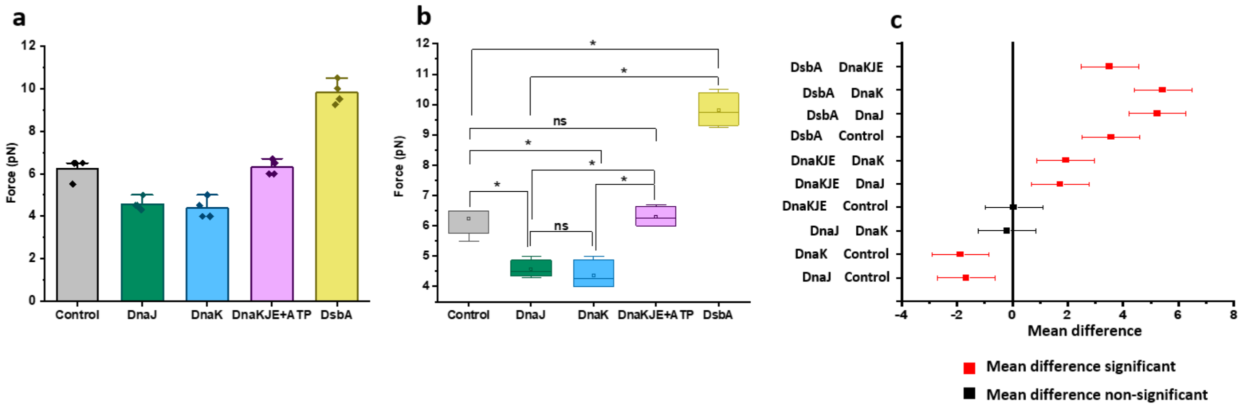

Supplementary Figure 14: Intersection force of talin WT-R3: (a) Intersection force. *Intersection forces of talin WT-R3 are measured both in the absence of any chaperones (control) and in the presence of the different chaperones. Intersection force is lowered in case of unfoldase chaperones such as, DnaJ and DnaK, while increases with the foldase such as, DsbA. Data points are calculated using four individual molecules. Error bars are represented as s.e.m.* ***(b) ANOVA analysis:*** *ANOVA analysis with different chaperones (n=4 molecules for each population) shows that intersection force for each chaperone population are statistically significant (R-sq.= 0.96) at *p≤0.05 level, with lower force in the presence of unfoldase chaperones and higher forces in the presence of DsbA (foldase). Since the DnaKJE complex promotes native folding ability in the substrate as in control, talin intersection forces are the same both in the control and with the DnaKJE complex, showing non-significant differences (ns). Similarly, unfoldases- DnaJ and DnaK also exhibit non-significant differences in the*

intersection forces. ***(c) Bonferroni post-hoc test:*** *Furthermore, we performed Bonferroni post-hoc test to check the pair comparison and observed that the means differences are statistically significant except control-DnaKJE pair and DnaJ-DnaK pairs.*

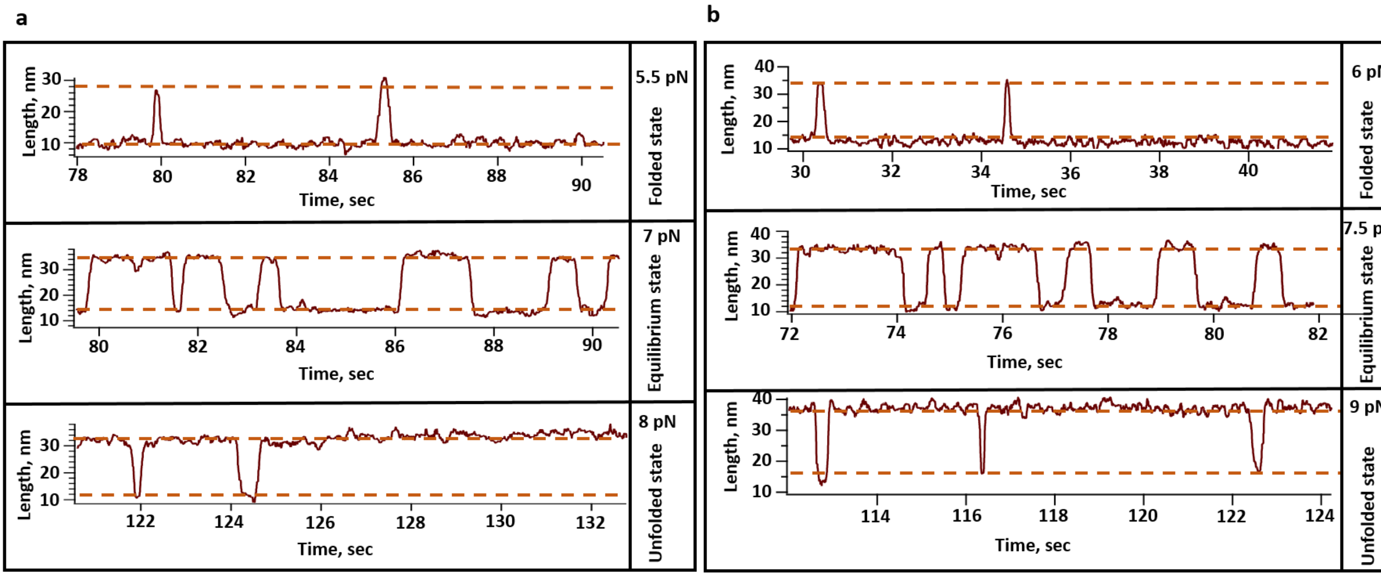

Supplementary Figure 15: Talin folding dynamics in presence of Hsp70 and Hsp40. (a) Hsp70: *In the presence of 3 µM Hsp70, talin folding dynamics has been observed to downshift to 7 pN force, with mostly unfolded state at 8 pN and folded state at 5.5 pN.* ***(b) Hsp40:*** *Similar to Hsp70, talin equilibrium state shifts to 7.5 pN force with 1 µM Hsp40 and it reaches mostly unfolded state 9 pN and attains mostly folded state at 6 pN.*

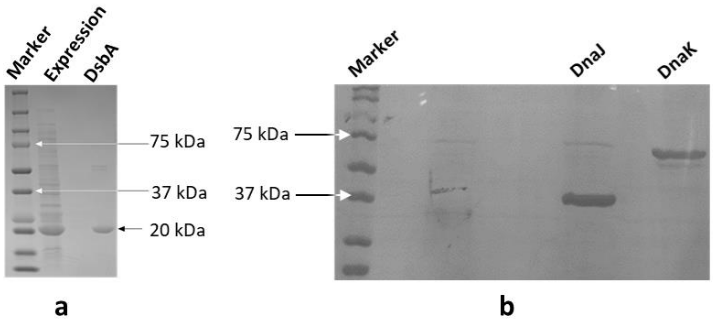

Supplementary Figure 16: SDS-PAGE of protein samples. *We have performed the SDS-PAGE of the SEC (size exclusion chromatography) purified protein samples.* ***(a)*** *We observed DsbA expression and its purification;* ***(b)*** *DnaK and DnaJ.*

Supplementary methods:

Force calibration method:

In magnetic tweezers, the applied force can be empirically calibrated by several ways, depending on the construct: either by measuring the lateral fluctuation for large construct (~10 µm) such as DNA or by relating protein unfolding extension to polymer elasticity models for smaller construct (~50 nm) (Mora et al., Chem. Soc. Rev., 2020). Since we are using small globular protein with step size with 6-15 nm, we sought to follow the calibration method for the protein construct. Thus, we have measured the unfolding extensions against the force in PBS buffer at 25ºC and fitted with the freely jointed chain (FJC) model of polymer elasticity (Supplementary Figure 17A) with the *Lc* value of 16.6±0.3 nm and *Lk* of 1.1±0.2 nm. These values are in well agreement with the previous publications (Popa et al., *J. Am. Chem. Soc*., 2016). To reconcile the accuracy of the force calibration, we fitted the force at varying magnet position by exponential magnet law (Supplementary Figure 17B) derived by Popa et al (Popa et al., *J. Am. Chem. Soc*., 2016).

We checked the sensitivity of force calibration by imposing the deviation values of protein L polymer such as, 0.3 nm for Lc and 0.2 for Lk and observed that the effect of these deviations is within the 95% confidence level. Due to the size heterogeneity of paramagnetic beads and difference in the tether point at the bottom pole of the beads, the calibrated force has ≤10% uncertainty (Chen et al., *J. Am. Chem. Soc*., 2015; Popa et al., *J. Am. Chem. Soc*., 2016. However, the M270 paramagnetic beads have lower coefficient of variation of ~2% than other beads of the provider, which could also ensure the negligible variation among beads. During the experiments, it is important to position the magnets by adjusting the voice coil in reproducible manner for the force application.

Testing the accuracy of force calibration:

We corroborated our force calibration method by analyzing the folding probability data of protein L molecule and then compared with the folding probability of protein L, reported by other authors who study protein nanomechanics by magnetic tweezers (Valle-Orero et al., *Angew Chem Int Ed Engl*. 2017; Valle-Orero et al., *J Phys Chem Lett*., 2017; Haldar et al., *Nat Commun*. 2017). The folding probability of protein L is highly force-dependent and we have monitored it within 4-11 pN force range. We observed that half-point force (defined as a force where FP=0.5) of protein L is ~8 pN force and it changes significantly with 1 pN force deviation. For example, at 7 pN, the folding probability can increase by 56% ($FP_{7\ pN}$= 0.78), while decreases by 72% at 9 pN ($FP_{9\ pN}$=0.14). Thus, slight deviation in force calibration certainly affects the folding probability of protein under force, by changing the applied force on the protein tether at a particular magnet position. Notably, in our case, we found protein L exhibit half-point force at ~8 pN force, which strongly coincides with that proposed by other groups and all the FP values are in well-agreement with their values (Supplementary Figure 17C). Therefore, this force calibration method has a precise detection at sub-piconewton range,

allowing us to observe the force-dependent folding dynamics, which strongly claims its fiduciary force measurement by our force calibration method. Lastly, the force calibration has also been confirmed by observing the conventional B-S overstretching transition of 565 bp dsDNA at ~66 pN, which is within the range of well-defined force standard of DNA B-S transition at 65 pN (Smith et al., *Science*, 1996). Although we have shown the detailed force calibration in our previous works; since these are not published yet, we have included them in this paper as well.

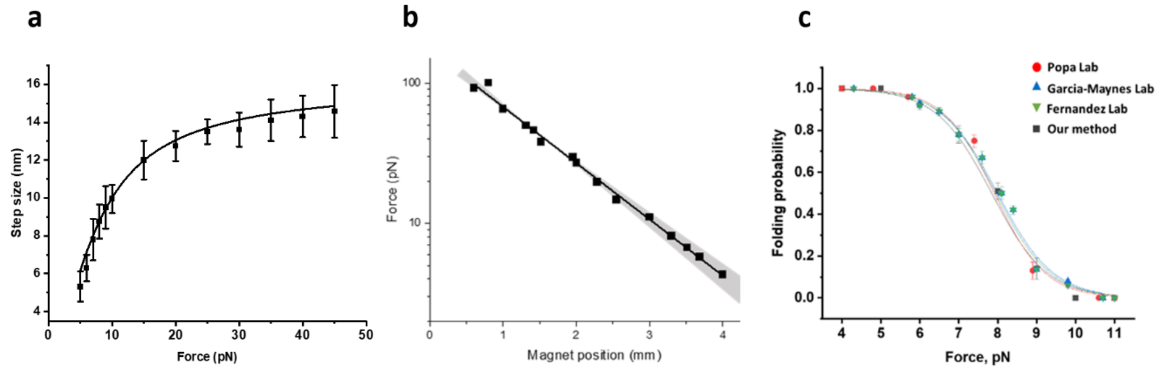

Supplementary Figure 17: Magnet law. *We calibrated the applied force using the exponential magnet law, proposed by Popa et al., JACS 2016 and demonstrates its accuracy with our instrumental set-up.* ***(a)*** *Protein L has been used as a protein force calibration and its step size or extension (~6-15 nm) are plotted as a function of force and fitted to the freely jointed chain (FJC) model of polymer elasticity.* ***(b)*** *Accuracy of the magnet law has been reconciled by plotting the applied force against the magnet position with shaded region at 95% confidence, which is also confirmed by conventional B-S overstretching transition of 565 bp of dsDNA at 66±1.2 pN.* ***(c)*** *Furthermore, the magnet law accuracy has been tested by analyzing the folding probability of protein L with the folding probability of protein L, studied by other groups and it has been observed to overlap closely with their data.*
