# Supplementary material for: Direct observation of chaperone-modulated talin mechanics with single-molecule resolution
Source: Commun Biol. 2022 Apr 4;5:307. doi: 10.1038/s42003-022-03258-3 (PMC8979947; doi:10.1038/s42003-022-03258-3)
Supplement: Supplementary file 3 — Description of Additional Supplementary Files [file 42003_2022_3258_MOESM3_ESM.pdf]

Description of Additional Supplementary Files

File Name: Supplementary Data 1

Description: Supplementary data (in excel format) for the graphs in the main and supplementary figures.
